# Barriers to hand hygiene compliance in intensive care units during the COVID-19 pandemic: A qualitative study

**DOI:** 10.3389/fpubh.2022.968231

**Published:** 2022-08-18

**Authors:** Maryam Ahmadipour, Mahlagha Dehghan, Mehdi Ahmadinejad, Maryam Jabarpour, Parvin Mangolian Shahrbabaki, Zahra Ebrahimi Rigi

**Affiliations:** ^1^Department of Pediatric, School of Medicine Afzalipour Hospital, Kerman University of Medical Sciences, Kerman, Iran; ^2^Department of Critical Care Nursing, Facullty of Nursing and Midwifery, Kerman University of Medical Sciences, Kerman, Iran; ^3^Nursing Research Center, Kerman University of Medical Sciences, Kerman, Iran; ^4^Department of Anaesthesiology, School of Medicine, Shahid Bahonar Hospital, Kerman University of Medical Sciences, Kerman, Iran; ^5^Clinical Research Unit, Shahid Bahonar Hospital, Kerman University of Medical Sciences, Kerman, Iran; ^6^Department of Nursing, School of Nursing and Midwifery, Iranshahr University of Medical Sciences, Iranshahr, Iran

**Keywords:** hand hygiene, intensive care units, qualitative study, healthcare workers, barriers, COVID-19

## Abstract

**Background:**

The practice of hand washing is an effective way to prevent contamination and disease transmission. Following the COVID-19 pandemic, hand washing has become increasingly important. Therefore, this qualitative study aimed to understand barriers to hand hygiene compliance among healthcare workers during the COVID-19 pandemic.

**Materials and methods:**

Twenty-five healthcare workers from intensive care units were sampled using purposive sampling in a qualitative content analysis study. Data were collected through a semi-structured interview and field notes. Based on the Lundman and Graneheim approach, the data were analyzed. COREQ checklist was used to report the research.

**Results:**

According to the findings, there are three main categories of barriers to hand hygiene practice: barriers related to individuals (including two subcategories of lack of knowledge of healthcare workers and healthcare workers' improper attitude), barriers related to management (including two subcategories of wrong behavioral patterns and unsuitable training and planning), and barriers related to organizations (including four subcategories of heavy workloads, improperly designed wards, a lack of equipment, and lack of quality equipment).

**Conclusions:**

This research indicates that hand washing practice increased during the COVID-19 pandemic. Nevertheless, some barriers persist, resulting in a decline in hand washing compliance among health care workers. This finding can help managers and policymakers remove barriers to hand washing compliance and improve healthcare workers' adherence to hand washing.

## Introduction

COVID-19 is a highly contagious disease that has spread rapidly throughout the world, beginning in Wuhan, China, at the end of 2019. It has affected more than 210 countries in its first wave (1). The path of transmission of COVID-19 facilitates its spread among people and renders all individuals susceptible to the disease (2). As a global public health concern, COVID-19 has been declared an emergency by the WHO (3). Extensive measures were recommended to reduce the spread of infection, including keeping a safe distance, covering the mouth and nose with a tissue when coughing and sneezing, washing hands frequently, and wearing masks (4). In order to prevent nosocomial infections, the WHO and the Centers for Disease Control and Prevention advise hospitals to follow infection control standards and precautions, such as hand washing and wearing personal protective equipment (4).

Hospital-acquired infections (HAIs), also known as nosocomial infections, presented a severe challenge to healthcare professionals worldwide during the COVID-19 pandemic (5). Nosocomial infections are associated with higher clinical costs, drug use, and hospital stays (6). Infection control (IC) practices can prevent and control hospital-acquired infections (7). Due to a lack of self-protection and containment measures, HAIs are increasing (5).

There are two types of IC: expanded precautions and standard precautions. All patients are provided with standard precautions, including hand hygiene (HH), handling bodily fluids, and preventing injury from sharp objects. Alternatively, expanded precautions are applied according to the mode of disease transmission, such as contact, droplet, and airborne transmission (8). Practicing hand hygiene in healthcare settings is crucial to preventing nosocomial infections (7).

Healthcare workers frequently contact patients, making it easy for microorganisms to be transmitted through their hands (9). In intensive care units (ICUs), the problem is more critical due to a higher infection rate than in other wards, as well as high-risk patients due to multiple injuries, low awareness, and weak prevention mechanisms (10). Hand hygiene compliance has improved patient health and safety and decreased complications, hospital stays, and death risks. Despite hand hygiene techniques being simple, individuals find them challenging to follow, and numerous studies have shown that healthcare workers have low acceptance and poor adherence to these practices (11).

In hospitals, hand hygiene is affected by some factors (12). A study found that nurses tend to adhere to hand hygiene less frequently due to high workloads (13). Researchers found that inadequate training in infectious disease control and prevention was one of the reasons for the rise in COVID-19 cases in hospitals (14). A study conducted in a hospital during the COVID-19 revealed that hand hygiene differed according to criteria and moment. Motivation, adequate human resources, supervision, and training are necessary to improve hand hygiene (15). Based on the results of a systematic review, there is a lower compliance rate with hand hygiene in intensive care units compared to other wards. Physicians have a lower compliance rate than nurses. Moreover, before contacting patients, healthcare workers exhibit a lower level of compliance than after contacting patients (16). Furthermore, another study found that hand hygiene is more commonly practiced during night shifts than morning shifts and patient protection than self-protection (10).

While this may be true, the pandemic allowed to change behavior through reactive stimuli such as fear and knowledge (increased infection rates), which led to greater compliance with hand hygiene practices (17); little information is available regarding hand hygiene compliance rates during the COVID-19 pandemic (17). Consequently, most studies have only been quantitative and have examined compliance with hand hygiene practices (9, 10). The behavior associated with HH was also complex and challenging to comprehend, clarify, or alter (18). It is important to use qualitative research methods to investigate individuals' beliefs, attitudes, experiences, and intentions (19). Therefore, this finding contributes to establishing a habit of hand hygiene rather than merely reacting to an incident.

As monitoring and controlling nosocomial infections is one of the essential measures in any hospital, understanding the barriers to hand hygiene can help improve hand washing compliance among healthcare workers. So, this approach was used to explore barriers to hand hygiene compliance in intensive care units during the COVID-19 pandemic as perceived by healthcare workers.

## Materials and methods

### Study design

This qualitative study applied the conventional content analysis method with a descriptive-explorative approach (20). A qualitative study is a critical tool for studying emotions, perceptions, and knowledge about the complexities of human reactions, which cannot be obtained *via* quantitative research. Content analysis is a systematic coding and categorizing method used to understand, analyze, and conceptualize the underlying concepts of qualitative data (21).

### Sample and setting

Twenty-five healthcare workers in the intensive care units of a teaching hospital participated in the study. The healthcare workers included anesthesiologists (*n* = 5), nurses (*n* = 18), and physiotherapists (*n* = 2). Shahid Bahonar Teaching Hospital is the largest trauma center in southeast Iran, with 350 beds and four intensive care units. Purposive sampling was used to select participants with maximum variation in age, gender, work experience, and education level. The method consisted of interviewing participants suitable for the study, including health care workers who served in intensive care units during the outbreak of the COVID-19 pandemic and were willing and able to share their experiences. These criteria were determined by asking health care workers. The inclusion criteria required healthcare workers with at least 6 months' experience in intensive care units and fluency in Persian. The study excluded participants with a history of mental illness. The sample size of a qualitative study depends on the saturation of the data (21), which determines whether sufficient data are present to form a comprehensive understanding (22). After interviewing 22 participants, the present study reached saturation, however, three additional interviews were conducted to confirm data saturation.

### Data collection procedure

Data were collected from late April to late May 2020 through semi-structured individual interviews with open-ended questions. Interviews were conducted by PM in each case. In the beginning, some prepared questions were asked to familiarize the researcher and create a friendly atmosphere with the participant. As part of the interview process, an interview guide was used. Afterwards, the interviews were focused on the study's purpose. Interviews varied from 45–55 min; during each interview, the researcher encouraged healthcare workers to participate in conversation and interaction and share their experiences. Some questions are as follows: “What facilities are available for hand hygiene practices in your ward? According to your beliefs, what are the five moments when hand hygiene should be practiced?” Please talk about your experience regarding barriers to hand hygiene during care provision?” As part of this study, field notes were used to collect data, and in all visits, the conditions of the ward and field observations were noted.

### Ethical considerations

This qualitative study is approved by the Ethics Committee of Kerman University of Medical Sciences with the code [No: IR.KMU.REC.1398.581]. After selection of the participants, the study objectives were explained to the participants and informed and written consent was obtained for audio recording at the beginning of the interview. Participants were ensured about the confidentiality of the data and the right to enter and withdraw from the study. Each participant was identified with a number. Interviews were conducted in person at a specified time and place in the health center.

### Data analysis

A content analysis methodology was employed in the analysis of the data following Graneheim and Lundman's five steps (23). In the first stage, each interview was transcribed immediately. The full texts of the interviews were read several times to immerse the researchers in the data and obtain a general perception of the content. Each interview text enters into MAXQDA software version 10 to manage the data. In the second step, the full texts of the interviews were read to determine the meaning units relevant to the aim of the study. In the third stage, meaning units were condensed and labeled with relevant codes. The initial codes were divided into subcategories based on similarities and differences in the fourth stage. One category contained similar manifest codes. Finally, the latent content in the data was extracted. During the data collection and analysis process, the researcher recorded any sparks related to the data and used them for subsequent interviews. PM and MJ analyzed the interviews. All extracted categories and themes were checked and approved by the authors. MA, MD, MA, PM, and ZE contributed to the composition, review, and correction of the final written report. Table 1 illustrates an example of the analysis process.

**Table 1:** An example of qualitative content analysis process.

**Category**	**Subcategory**	**Code**	**Meaning unit**
Organizational barriers	Heavy workload	Emergency care of an ICU patient Fatigue following intensive care of critically ill patients High workload of staff Impossibility of keeping away from critically ill patients Simultaneous care of two patients Fatigue following night shifts High number of patients	Many times, I did not have enough time to wash or disinfect my hands due to the high workload in the intensive care unit and the emergency of some procedure. When I have to take care of several patients at the same time especially in the night shift, I am less concerned about hand hygiene practice due to fatigu.

The Guba and Lincoln's criteria, including credibility, dependability, transferability, and confirmability, were used to determine trustworthiness (24). Credibility of data was achieved in various ways; the researchers were engaged in the field and at the site for long periods of time. Peer review was conducted by assessing background information, data collection methods, process, data management, transcripts, data analysis, procedure, and research findings. Several data collection methods were used to obtain supporting evidence, including semi-structured individual interviews and observations in the field. Moreover, participants were asked to participate in a member check during which they reviewed a short report of the analyzed and interpreted data. We did this in order to verify that the results were representative of their experiences and attitudes. By outlining all steps of the data collection process, context, the analysis, and direct quotes from participants, the transferability of the findings was ensured. Furthermore, maximum variability of sampling was considered.

### Findings

This study involved 25 healthcare workers working in ICUs with a mean age of 36.44 years and a mean work experience of 11.32 years. The healthcare workers included anesthesiologists (*n* = 5), nurses (*n* = 18), and physiotherapists (*n* = 2). Participants included two men and 23 women. Four of the twenty-nine potential participants declined to participate in the interview due to inadequate preparation and limitations imposed by the COVID-19 pandemic, resulting in a response rate of 86.2% (25/29). Based on the analysis of the data, eight subcategories, three categories, and a main category of “barriers to hand hygiene practice” have been identified. A summary of the main categories, categories, and subcategories is presented in Table 2.

**Table 2:** Main category, categories, and subcategories of barriers to hand hygiene practice in ICU healthcare workers.

**Main category**	**Categories**	**Subcategories**
Barriers to hand	Individual barriers	Lack of knowledge of healthcare workers
hygiene practice		Healthcare workers' improper attitude
	Management	Wrong behavioral patterns
	barriers	Unsuitable planning and training
	Organizational	Heavy workload
	barriers	Improperly designed wards
		The lack of equipment
		Lack of quality equipment

### Main category: Barriers to hand hygiene practice

Based on the analysis of healthcare workers' experiences, three subcategories were identified, including barriers associated with individuals, management and organizations, which we will address in the following paragraphs.

### Category 1: Individual barriers

According to the participants, improper attitudes of healthcare workers and inadequate knowledge of healthcare workers were two barriers related to knowledge and attitude. So, sufficient knowledge of healthcare workers about nosocomial infections and direct and indirect transmission of infectious agents played an essential role in obeying hand hygiene. In addition, by strengthening a positive attitude toward hand hygiene practice and convincing individuals that their behaviors will significantly impact the behavior of other colleagues, individuals' positive attitudes can be led to more adherence to hand hygiene compliance by healthcare workers.

### Subcategory A. lack of knowledge of healthcare workers

In most cases, the participants' experiences indicate that healthcare workers are unaware of the consequences of poor hand hygiene practices, including antibiotic resistance, hospital stay length, nosocomial infections, and even mortality. The absence of awareness of staff, particularly novices, contributed to non-compliance with hand hygiene. Due to the absence of apparent contamination of the hands during the care provision or the use of substitute gloves for hand hygiene, participants felt that hand hygiene was not necessary, which led to less hand hygiene compliance.

“*Sometimes we do not take hand hygiene seriously because we do not know enough about the complications of poor hand hygiene practice.” (P1-A nurse)*.

“*Despite knowing the five moments when hand hygiene should be practiced, I did not fully practice it because I didn't know its importance.” (P5-A nurse)*.

### Subcategory B. healthcare workers' improper attitude

Most participants' experiences revealed that healthcare workers' negative beliefs and attitudes toward hand hygiene practice played an essential role in non-compliance with hand hygiene because of despondency, lower productivity, lower enthusiasm, and low confidence. On the other hand, one aspect of healthcare workers' attitudes is their impact on other colleagues on the ward. Thus, a negative attitude toward hand hygiene could significantly impact the behavior of other healthcare workers, resulting in less compliance with hand hygiene practices.

“*Often, we do not practice hand hygiene because we do not believe in the importance of hand hygiene and do not get used to it. We would practice hand hygiene more if they reported monthly statistics of nosocomial infections and their complications.” (P2-A nurse)*.

“*My hand washing habits became less frequent after the COVID-19 pandemic since I didn't care about it.” (P9-An anesthesiologist)*.

### Category 2: Management barriers

In most cases, the participants addressed the wrong behavioral patterns of supervisors and improper planning and management training. Wrong behavioral patterns hamper the pace of recovery and growth in one place. It is necessary to recognize toxic behavioral patterns before deciding on ways to halt and change them. In social settings, it is crucial that people observe the principles, rules, and guidelines that govern those settings and engage in positive patterns and norms. In addition, managers can promote hand hygiene practices through proper planning, training, and monitoring.

### Subcategory A. wrong behavioral patterns

Most participants' experiences demonstrated that healthcare workers mimicked the wrong behavioral patterns of managers as head nurses or in charge and doctors as superiors or heads of departments. Consequently, the lack of adherence to hand hygiene compliance by colleagues, managers, and doctors affected other healthcare workers' performance.

“*Doctors, residents, and head nurses, who can be good role models, do not often pay enough attention to hand washing practice. Therefore, we underestimate the importance of hand hygiene practice and do not comply with it properly.” (p6-A nurse)*.

“*As I often see my colleagues or doctors not complying, I don't do it because they don't.” (p4-A nurse)*.

### Subcategory B. unsuitable planning and training

In most cases, the participants' experiences revealed that inadequate training led to healthcare workers not having a clear picture of what is expected of them at work. Therefore, they will have difficulty performing tasks, including hand hygiene practice. In addition, managers' ineffective planning, poor monitoring, not providing positive feedback, and insufficient support have affected healthcare workers' adherence to hand hygiene. As well, managers did not pay enough attention to the problems and barriers to hand hygiene practices that contributed to poor hand hygiene. Participants considered insufficient management control over the evening and night shifts and inappropriate microbial culture to be administrative barriers to hand hygiene practice.

“*I easily neglect my hand hygiene because there is no positive culture for hand hygiene and managers do not pay attention to culture building regarding it.” (p14-An anesthesiologist)*.

“*There is less supervision during evening and night shifts, so hygiene protocols such as hand washing are less likely to be observed.” (p22-A nurse)*.

### Category 3: Organizational barriers

A number of participants have addressed heavy workloads, poor design of hospital wards, inadequate equipment, and low-quality equipment in this regard. Hand hygiene practices are hindered by the high workload of ICUs. Additionally, changes to the physical design of hospital wards may promote proper hand hygiene. Ample and qualified equipment on the wards, especially in ICUs, will increase hand hygiene practice and reduce poor hand hygiene complications.

### Subcategory A. heavy workload

Based on the experiences of several participants, the high workload in ICU is an essential barrier to hand hygiene. The reason is stress, a lack of peace of mind, and the rush to finish the tasks assigned. They mentioned that during the handling of many patients, caring for two patients simultaneously, hand hygiene practice is inevitably forgotten. They also noted that the impossibility of keeping away from critically ill patients was another barrier to hand hygiene practice. Moreover, hand hygiene practice was also impossible for emergency patients who need intensive care in critical situations. In addition, fatigue due to overwork in the ICU and night shifts prevented them from practicing proper hand hygiene.

“*When I have to take care of several patients simultaneously, especially on the night shift, I am less concerned about hand hygiene practice due to fatigue.” (p14-A nurse)*.

“*There are so many patients that I become tired and don't follow handwashing protocols any longer.” (p8-A nurse)*.

### Subcategory B. improperly designed wards

Based on the experiences of several participants, working in non-standard environments was one of the factors that prevented them from practicing hand hygiene effectively. In some situations, for instance, the lack of accessible handwashing sinks and the distance between the sinks and the patients' beds make hand hygiene practice difficult due to inconvenience and dissatisfaction. Many participants indicated that the health system infrastructure was inefficient with regard to hand hygiene practices. As a result, handwashing sinks and reducing the distance between sinks and patients' beds will facilitate access and promote better hand hygiene practices.

“*Many times, there was no sink when I wanted to wash my hands.”* (p16-A physiotherapist).

“*Patient's bed is far from the toilet.”* (p3-A nurse).

### Subcategory C. the lack of equipment

In light of the experiences of many participants, the absence of sinks and smart faucets for hand washing was one of the barriers to hand hygiene. Many participants noted that they were unable to dry their hands due to the lack of tissue paper and a hand dryer, causing poor hand hygiene. Other barriers to hand hygiene practices included a lack of detergents or personal protective equipment, a lack of skin moisturizers after hand washing, and insufficient funds to purchase hand washing equipment.

“*Often, I have neglected hand hygiene due to the lack of tissue papers for drying my hands.”* (p9-A nurse).

“*A few gloves, disposable towels, and disinfectant solution are available.”* (p15-A nurse).

### Subcategory D. lack of quality equipment

In most cases, the participants' experiences revealed that poor quality equipment reduces hand hygiene practices. A study noted that the poor quality of soap and disinfectants for hand hygiene resulted in skin dryness and itching. This led to inadequate hand washing by healthcare workers.

“*I have not done hand rub for a month because of the poor quality of the disinfectants and the allergy I felt after using them.” (p23-A nurse)*.

“*I washed my hands less often due to extreme dryness and sensitivity caused by hand sanitizers.” (p12-A nurse)*.

## Discussion

In the context of the COVID-19 pandemic, this qualitative study explored nurses' experiences with hand washing compliance barriers in ICUs. This study showed that despite increased compliance with hand hygiene practices due to the COVID-19 pandemic, healthcare workers in intensive care units face several obstacles to hand hygiene practices. Based on the analysis of healthcare workers' experiences, it was discovered that the main categories of “barriers to hand washing practices” consisted of three categories: individual, manager, and organizational barriers.

The lack of awareness of healthcare workers led to poor hand washing practices. Some healthcare workers were unaware of the importance of hand washing and its role in decreasing nosocomial infections and its costs and problems. Also, some healthcare workers did not believe in hand hygiene and were inattentive to it. In contrast, in the present study, (25) lack of obvious contamination on the hands, substitute of gloves for hand hygiene, as barriers to hand washing practice (25). Some studies have shown that healthcare workers have sufficient knowledge about hand washing practices and believe that unclean hands are an essential route of cross-infection in hospitals (26, 27). In addition, (28) believed that non-compliance with hand hygiene was not necessarily related to the knowledge of healthcare workers. Also, the staff was aware of the importance of hand washing, but they did not practice it (28). Despite the COVID-19 pandemic increasing healthcare workers' adherence to hand hygiene practices, compliance has declined over time due to poor understanding of procedures and a lack of educational interventions to recognize hand hygiene opportunities. Additionally, this may result from an inadequate appraisal of the essential issues of hand washing compliance, resulting in inadequate awareness among healthcare workers and poor hand washing practices. A lack of academic training is another factor that contributes to a reduction in the level of knowledge of health care workers, especially novices. Consequently, training healthcare workers on proper hand washing methods with reminder posters can significantly improve their awareness of and knowledge of hand hygiene (29).

The wrong attitude of healthcare workers toward hand washing practice led to less adherence to hand hygiene compliance. Some studies have shown that the positive attitude of healthcare workers has been associated with an increase in hand washing practice (30, 31). Although the COVID-19 pandemic prompted personnel to increase their hygienic practices. After a COVID-19 disease has subsided, healthcare workers may be less inclined to wash their hands due to a lack of positive attitudes toward hand washing, behavioral beliefs such as feeling happy after hand washing, and a lack of evaluation of behavioral outcomes, such as the value of self-care and family care. Consequently, designing educational programs will play an essential role in increasing healthcare workers' attention and positive attitude regarding hand washing practices and standardizing health behaviors to increase hand hygiene (32).

The wrong behavioral patterns prevented hand washing practice. Similar to the present study, Studies addressed that the behavior of physicians, especially chief physicians, played an essential role in their compliance with hand hygiene standards by other people (31, 33, 34) also reported that role modeling plays a vital role in following hand hygiene standards (34). This issue emphasizes the crucial role of senior hospital staff in promoting hand washing practices and improving patient safety, in addition to the driving role of the COVID-19 disease. Because senior hospital staff plays a lasting and permanent role, the support and involvement of senior hospital staff, including physicians, may assist in removing barriers to hand washing practice. The fundamental concepts of behavioral patterns of hand hygiene must, however, also be emphasized in order to change individual attitudes toward hand hygiene (35).

Barriers related to improper management and planning were identified as essential barriers to hand hygiene practice (36) believed that hospital authorities were responsible for ensuring proper hand washing practices and should have more control over barriers to remove them (29). Therefore, training sessions, positive feedback, managerial support, and a proper environment were recommended to promote hand washing (25). The COVID-19 disease demonstrates the need to change management strategies for improving infection control (IC) practices, including adherence to hand hygiene by healthcare workers.

Participants in the present study considered heavy workload, fatigue, emergency patients who need intensive care in critical situations, and many patients as the main reasons for non-compliance with hand hygiene (37) believed that high workload and high patient-to-nurse ratio caused hand washing compliance to be forgotten or even impossible (37). Also, numerous studies have identified workload as one of the barriers to healthcare workers' compliance with hand hygiene (25, 38). In addition, several studies have shown that healthcare workers believed that they did not have enough time to perform hand washing in emergencies (27, 36, 39) showed that healthcare workers were less concerned about hand washing practice at the end of their shift work due to fatigue, and the longer the rest period between shift work, the higher hand hygiene was practiced (39). As a result, although the healthcare workers are familiar with the correct hand washing technique, they will not be able to practice hand hygiene due to the heavy workload that multiplied during the COVID-19 pandemic. Moreover, a management system capable of handling emergencies, adjusting high workloads, and increasing hand hygiene is necessary.

Improper design of the physical space of the ward was mentioned as some of the obstacles to hand washing practice (25) considered high workload, limited hospital space, and unavailability of sinks as barriers to hand washing practice (25), which were consistent with our study. As a result of a lack of space, the COVID-19 ICUs are located in departments without facilities for washing hands, resulting in poor handwashing practices.

Lack of equipment, on the one hand, and poor-quality equipment, on the other hand, were Also mentioned as significant reasons for hand hygiene non-compliance (40) demonstrated that the staff had sufficient knowledge and readiness to comply with hand hygiene. Unfortunately, the lack of appropriate equipment and facilities prevented them from following recommended hand hygiene protocols (40). To remove barriers and increase compliance with hand hygiene protocols, appropriate cleaning materials such as soap, paper towels, tissue papers, smart faucets, and the availability of hand washing sinks were also considered (41, 42) believed that the main reasons for not practicing hand hygiene were the lack of good hygiene products, insufficient tissue papers, lack of hand dryers, and repeated washings causing skin damage (42). At the beginning of the COVID-19 outbreak, hospitals faced a severe shortage of disinfectants due to the lack of prior forecasting, which adversely affected the observance of hand hygiene. The hospitals purchased low-quality disinfectants because of the dire need for disinfectants, which decreased staff adherence to hand washing, especially after the COVID-19 pandemic subsided. As a result of providing adequate and high-quality equipment, skin damage can be reduced, hand hygiene can be ensured, and cross-infection can be prevented. Therefore, it appears that management's attention promotes handwashing.

## Limitation and strengths of the study

The small sample size and the fact that the study took place in a trauma center were among the limitations of the present study. Therefore, generalizations should be made with caution. A small number of participants was present in some groups, which may make it difficult to differentiate between professional groups. Also, The COVID-19 pandemic constraint caused some potential participants to decline to participate in the interview. Notwithstanding these limitations, we gained insight into the conclusion that despite scientific evidence that improved hand hygiene had resulted in a reduction in hospital infections during the COVID-19 pandemic and that pandemic conditions induced personnel to wash their hands more frequently, modifying individual, managerial, and organizational barriers is the feasible solution that can be useful for future research, education, and practice.

## Conclusion

Several factors contribute to non-compliance with hand hygiene practices, including lack of knowledge, incorrect behavior patterns, insufficient training, heavy workloads, poorly designed wards, and low-quality equipment. Providing color-coded reminder boards, ensuring easy access to washrooms, monitoring disinfection solutions, and providing training and scientific information regarding the importance of improving hand hygiene can remove hand washing barriers. Standard precautions during COVID-19 reduced HAIs, but employee adherence to hand hygiene declined as the disease subsided; therefore, the hospitals must pay attention to barriers preventing employees from complying.

## Data availability statement

The original contributions presented in the study are included in the article, further inquiries can be directed to the corresponding author.

## Author contributions

PM, MD, MeA, and MaA supervised this study and designed the study. PM and MD prepared the interview guide and analyzed the interviews. MJ conducted the interviews. PM, MD, and ZE cooperated in composing, reviewing, and correcting the written version. The research team read and confirmed the final version. All authors contributed to the article and approved the submitted version.

## Conflict of interest

The authors declare that the research was conducted in the absence of any commercial or financial relationships that could be construed as a potential conflict of interest.

## Publisher's note

All claims expressed in this article are solely those of the authors and do not necessarily represent those of their affiliated organizations, or those of the publisher, the editors and the reviewers. Any product that may be evaluated in this article, or claim that may be made by its manufacturer, is not guaranteed or endorsed by the publisher.
